# Psychological Intervention Program to Control Stress in Youth Soccer Players

**DOI:** 10.3389/fpsyg.2019.02260

**Published:** 2019-10-16

**Authors:** Aurelio Olmedilla, Isabel Mª Moreno-Fernández, Verónica Gómez-Espejo, Francisco Javier Robles-Palazón, Isidro Verdú, Enrique Ortega

**Affiliations:** ^1^Department of Personality, Evaluation and Psychological Treatment, Sports Activities Service, Campus of Excellence Mare Nostrum, University of Murcia, Murcia, Spain; ^2^Department of Basic, Evolutionary and Educational Psychology, Autonomous University of Barcelona, Barcelona, Spain; ^3^Department of Psychology, Real Murcia C.F., Murcia, Spain; ^4^Department of Physical Activity and Sport, Campus of Excellence Mare Nostrum, University of Murcia, Murcia, Spain; ^5^Department of Computing and Systems, Campus of Excellence Mare Nostrum, University of Murcia, Murcia, Spain

**Keywords:** psychological training, adolescent, football, stress, psychological skills

## Abstract

The influence on the psychological well-being of the players and their sports performance seems to be one of the keys to the current sports practice. The purpose of this study was to determine the effectiveness of a psychological intervention program for stress control in youth soccer players. A total sample of 19 male youth soccer players (age: 16.3 ± 0.99 years; years playing soccer: 10.89 ± 1.56 years) completed the current research. The Psychological Characteristics Questionnaire related to Sports Performance (CPRD) was used to assess stress factors related to sports competition. A program based on Cognitive-Behavioral Therapy was implemented during eight sessions of approximately 50 min each. A pre-post design was used and statistical differences between pre- and post-measures were checked through dependent sample *t*-tests. The results indicated that the post-test scores were higher than the pre-tests in “Influence of the Evaluation of Performance” and “Mental Skills” factors, which supposes a significant improvement of the stress management related to performance evaluation, as well as the use of psychological resources and techniques. In addition, the post-test scores were also higher in the “Stress Control” factor, although in this case the differences were not statistically significant. Practical indications deriving from the findings of this study can help youth soccer players to manage the stress of competition using a psychological training program.

## Introduction

Currently, psychological interventions are usually utilized in sport, thanks to their positive influence on the psychological well-being (Golby and Wood, 2016; Breslin et al., 2017) and sport performance (Brown and Fletcher, 2017; Gross et al., 2018). Psychological training can help several psychological variables such as motivation, concentration, self-confidence, or activation level (Beckmann and Elbe, 2015; Olmedilla and Domínguez-Igual, 2016), as well as the acquisition of psychological skills as techniques and resources to manage the sport practice (Simonsmeier and Buecker, 2017; McCormick et al., 2018).

In sports with changeable demands where it is necessary to make complex decisions continuously (team sports), cognitive skills have equal or even more relevance than technical or tactical executions (Escolano-Pérez et al., 2014; Larkin et al., 2018; Olmedilla et al., 2018a). Although psychological skills do not provide an increment of the athlete’s sport performance on their own, they can assist athletes (in conjunction with the physical, technical, and tactical training) with the achievement of higher level of performance (Abdullah et al., 2016). For instance, the knowledge of the psychological influence might help an individual to apply appropriated interventions for neutralizing some factors that could obstruct the sport performance (Gimeno et al., 2007). Thus, variables such as motivation, concentration, stress control, or self-regulation of mood have been proposed as key to explain differences in athletes’ sport performance (Cerasoli et al., 2014; Mercado et al., 2017; Swann et al., 2017).

Psychological training is not only important in professional or elite sport, but also in grassroots sports. Young athletes sometimes need a certain amount of motivation to obtain a good level of adherence to sport practice (matches and trainings), and require efficacy resources to manage the stress derived from competitions. A suitable psychological development in young athletes will increase their achievement of goals and satisfaction in sport (Navarrón et al., 2017; Simonsmeier and Buecker, 2017; Brière et al., 2018), and will make easier the process of socialization through sport practice, managing better the requirement and pressure habitually supplied by coaches and/or parents (Tjomsland et al., 2016; Gómez-Espejo et al., 2017; Lorenzo et al., 2018).

Several stress sources exist at within youth sport practice, and their consequences may be really negative for the young athlete: less sport performance (Romero et al., 2010), absence of satisfaction, mental disorders (Schinke et al., 2018), dysfunctional attitudes (Fenoy and Campoy, 2012), sport dropout (Gimeno et al., 2007), or sport injuries (Ivarsson et al., 2017), among others. Likewise, stressful conditions during competitions provoke psychological disorders, such as loss of attentional focus or anxiety increase, that may negatively affect the athlete’s sport participation (Bennett and Maynard, 2017; Brown and Fletcher, 2017). Something we perceive as a threat is stressful and, therefore, produces significant changes in physiological, psychological, and behavioral responses; in a competitive sport context, this causes the athlete to think and act differently in stressful situations (Márquez, 2006). However, stress can also have positive connotations, helping the athlete to be prepared for the competition and favoring motivation, attention, and, consequently, the subsequent athlete’s sport performance (Ferreira et al., 2002; McCormick et al., 2018).

Properly managing stress is very important for any athlete since it entails directing the stressors in order to avoid the incorrect development of the sport activity (Kerdijk et al., 2016; Randall et al., 2018). Thus, this ability to control stress is one of the main requirements to achieve sport success, and athletes use different coping styles according to their individual characteristics (Fletcher and Sarkar, 2012; Kerdijk et al., 2016; McCormick et al., 2018). Coping might be defined generally as the cognitive and behavioral effort that is carried out by the athletes with the aim of controlling some demands (internal or external) that are really difficult to deal with using their own resources (Pinto and Vásquez, 2013; Nicholls et al., 2016; Arnold et al., 2017). Coping styles have been divided in two types, principally: problem-focused coping and emotion-focused coping, depending on whether the individual typically exerts cognitive and behavioral efforts to change a situation or typically adopts strategies to regulate any emotional distress, respectively (Urra, 2014; Nicholls et al., 2016). Scientific literature highlight the importance of stress control for an athlete’s sport performance and mental health (Gimeno et al., 2007; Schinke et al., 2018); so, stress management training programs seem to be an essential approach to sportspeople in both youth and professional levels.

Some authors defend the need to know the psychological profile of the athlete (Pazo et al., 2012) as a starting point to design specific psychological training programs that favor the optimal development of a sports career. Psychological training is another way of sports training that directly affects athletic development; this training must be based, on the one hand, on the learning of psychological skills and strategies that allow the most appropriate coping of different sports situations (Reyes et al., 2012; Portenga et al., 2017; Gross et al., 2018) and, on the other hand, on the promotion of the psychological well-being of the athlete that allows him/her to grow and mature as a person (Romero et al., 2010; Golby and Wood, 2016; Breslin et al., 2017; Olmedilla et al., 2018b). In any case, the psychologist must pursue that the athlete has a better expertise of his skills and psychological strategies, as well as the processes of reflection and decision-making in the different situations of sport and extra-sport (Olmedilla et al., 2018b).

The evaluation of psychological skills can allow working hypotheses about the most appropriate psychological intervention to favor sports performance (Olmedilla et al., 2010; Abenza et al., 2014). The knowledge of the psychological profile of an athlete allows to understand him/her better, improve communication processes with him/her, and increase the effectiveness of training (McCormick et al., 2017; Olmedilla et al., 2018a). Although it is not possible to find two equal athletes, there are certain common characteristics that lead to sport success. The weight of psychological factors in the definition of the successful athlete is high, so nowadays in sport, mental preparation and psychological skills can distinguish the successful athlete from the rest (Bahrololoum et al., 2012).

Therefore, the main purpose of the present study was to determine the efficacy of an intervention program for the acquisition of psychological skills to control stress in male youth soccer players.

## Materials and Methods

### Participants

A total of 19 male youth soccer players completed the current study (age: 16.3 ± 0.99 years; years playing soccer: 10.89 ± 1.56 years). All of them belonged to the same Spanish soccer club that was engaged in a Regional Amateur Soccer League of the Spanish Soccer Federation, and participated in four training sessions and one competitive match per week.

### Measures

Psychological variables were assessed using the Psychological Characteristics Related to Sport Performance Questionnaire (CPRD, Gimeno et al., 2001), based on the Psychological Skills Inventory for Sports (PSIS, Mahoney et al., 1987; Mahoney, 1989). The questionnaire consists of 55 items graded in a 5-option Likert scale (from totally disagree to totally agree) and grouped into five subscales: Stress Control (SC), Influence of Performance Evaluation (IPE), Motivation (M), Mental Skills (MSK), and Team Cohesion (TCOH), showing acceptable values of internal consistency for the total scale (*α* = 0.85) and for most of the subscales (αSC = 0.88; αIPE = 0.72; αM = 0.67; αTCOH = 0.78; αMSK = 0.34). According to the authors, the low internal consistency of MSK is probably related to it tapping a wide range of different skills but authors still keep the subscale due to the factorial loads shown by the items of this factor.

SC consists of 20 items and refers to athlete’s responses to potentially stressful situations and other training and competition demands. Higher scores denote the athlete has management skills to cope with sport-related stress. IPE consists of 12 items and refers to athlete’s responses to situations in which he/she or people close to him/her judge his/her performance. It also includes an assessment about antecedents of athlete’s performance judgment. Higher scores mean the athlete can control the impact of performance judgment. M consists of eight items referring to basic motivation to sport performance and achievement, as well as to the regular training and competition activities. Higher scores indicate strong motivation and commitment to competitive sport practice. MSK consists of nine items and assesses the use of different mental skills that are related to sport performance. Higher scores express better psychological resources to improve his/her performance. TCOH includes six items and assesses the extent to which the athlete feels attracted to and identified with the sport group. This scale has not been used in this study due to the nature of the target sports.

### Procedure

After the authors’ institution IRB approval (UM1551/2017), athletes were contacted through the psychological staff belonging to the club, who collaborated with the researchers to explain to coaches, parents, and athletes about the aims of the study and use of the information. Those who voluntarily agreed to participate in the current research signed an informed consent form (parents and athletes).

Subsequently, a psychological intervention program was implemented, whose theoretical framework was based on Cognitive-Behavioral Therapy and its four key principles (McArdle and Moore, 2012). This psychological training program was carried out in eight sessions of approximately 50 min each. All psychological intervention sessions were developed in small groups and before the regular soccer training practices, aiming to avoid the fatigue effect. The structure of the psychological session was based on previous therapy programs used in similar cohorts of athletes (Beswick, 2001; Dosil, 2006; Olmedilla and Domínguez-Igual, 2016). Table 1 shows the structure of the program with the number and content of the different intervention sessions.

**Table 1:** Chronogram and general contents of the program.

Sessions	Contents
Session 1	Initial assessment. Explanation of the procedure to be followed throughout the sessions. CPRD pre-test
Session 2	Motivation (I). Psychoeducation: explanation of the concept, types and ways to increase.
Session 3	Motivation (II). Setting objectives. Distinction between short-, medium-, and long-term objectives. Distinction between performance objectives and outcome objectives. How to carry out the registration of the objectives table.
Session 4	Attention-Concentration (I). Psychoeducation: explanation of the concept, types of attention, ways to increase attention and concentration.
Session 5	Attention-Concentration (II). Visualization. What is the visualization technique and how to apply it. Observation of testimonies of athletes who practice this technique.
Session 6	Activation level (I). Psychoeducation: explanation of the concept and how to increase or decrease the level of activation.
Session 7	Activation level (II). Relaxation. Explanation of what Jacobson’s progressive relaxation technique consists of and how to carry it out.
Session 8	Final assessment. CPRD post-test. Psychological Preparation Evaluation Questionnaire.

### Data Analysis

Prior to statistical analysis, the normal distribution (*p* > 0.05) of raw data set was checked using the Kolmogorov-Smirnov test. Descriptive statistics including means and standard deviations were calculated. Dependent sample *t*-tests were carried out to assess differences between the pre-intervention and post-intervention measures. Finally, effect sizes were calculated using the method previously described by Cohen (1988). All the analyses were completed using the statistical software SPSS version 21 (SPSS Inc., Chicago, IL, USA). Statistical significance was set at *p* < 0.05.

## Results

Table 2 shows the data obtained from the dependent sample *t*-test and the statistical significance in each of the CPRD subscales.

**Table 2:** Differences between pre- and post-intervention scores in each of the CPRD subscales.

	*M*	SD	SE	95% CI		*t*	df	Sig.	Cohen’s *d*
				Lower	Higher				
SC pre SC post	−2.684	6.377	1.463	−5.758	0.389	−1.835	18	0.083	−0.234
IPE pre IPE post	−2.631	4.867	1.116	−4.977	−0.285	−2.357	18	0.030	−0.389
M pre M post	−0.315	3.056	0.701	−1.157	1.788	−0.450	18	0.658	0.077
MSK pre MSK post	−2.263	4.201	0.963	−4.288	−0.238	−2.348	18	0.030	−0.788
TCOH pre TCOH pre	0.789	3.675	0.843	−0.982	2.560	0.936	18	0.362	0.291

*SC, stress control; IPE, influence of performance evaluation; M, motivation; MSK, mental skills; TCOH, team cohesion*.

**Figure 1:**
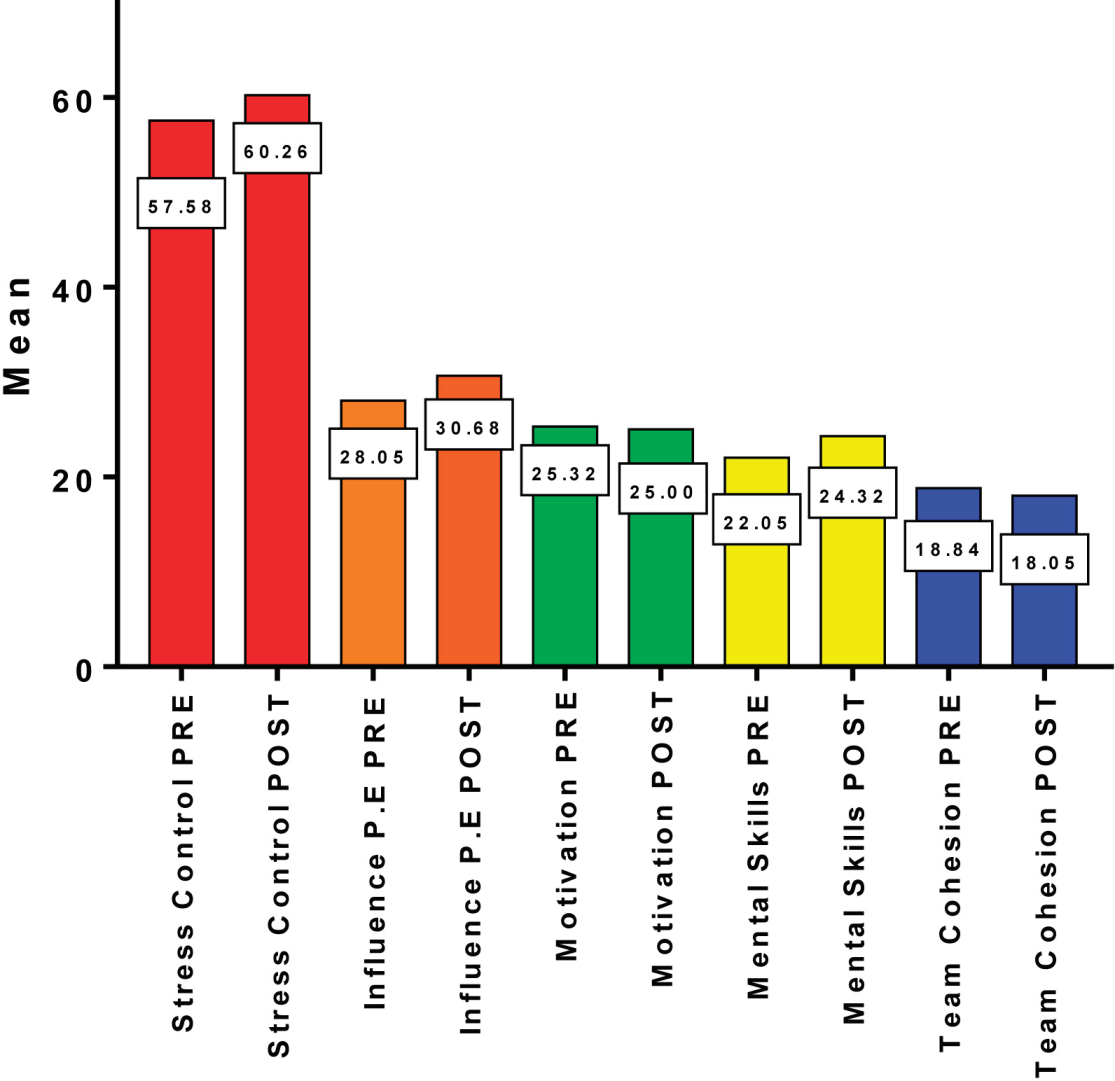
Graphical comparison of the total means pre-test and post-test.

Figure 1 presents the pre- and post-scores for each of the CPRD scales (SC, IPE, M, MSK, and TCOH). Statistically significant differences are found in two of the five factors: Influence of the Performance Evaluation (*p* = 0.030; *d* = −0.389) and Mental Skills (*p* = 0.030; *d* = −0.788); and there is a marginal significance in Stress Control (*p* = 0.083; *d* = −0.234). The results are complemented by a calculation of the effect size in order to evaluate the degree of change observed in the sample.

## Discussion

The aim of this study was to determine the effectiveness of a psychological intervention program (Cognitive-Behavioral Therapy) in a cohort of male youth soccer players for the acquisition of psychological skills to control and manage stress. The results indicated a general improvement in the scores of the players after the intervention program; thus, differences in the IPE and the MSK factors appeared statistically significant, which suppose an enhancement to stress management related to the evaluation of performance, as well as to the use of resources and psychological techniques. Likewise, scores after the program were also better in the SC factor, although in this case the differences were not statistically significant.

The results of the present study are similar to those obtained in recently published research (Aoyagi et al., 2017; Brown and Fletcher, 2017) and show the efficacy of psychological intervention programs both for the acquisition and learning of psychological techniques, and for the application of these improving skills for managing the stress of competition and sports practice, which could improve the psychological disposition of players favoring the increment of sport performance (McCormick et al., 2017). This psychological disposition, focused on variables such as motivation, concentration, or self-efficacy, will be optimized through the use of visualization, goal setting, or relaxation through breathing techniques, among others.

In addition, stress control has proven its usefulness and effectiveness in the field of athlete’s health, both physical and psychological. In terms of physical rehabilitation or prevention of sport injuries (Mankad and Gordon, 2010; Gagnon et al., 2015; Wesch et al., 2016; Olmedilla et al., 2017), there are numerous studies that correlate stress to the increase in the incidence of injury in sport. Ivarsson et al. (2017) found strong associations between responses to stress and the history of stressors with injury rates, results that agree with many other investigations (Johnson and Ivarsson, 2011; Edvardsson et al., 2012). Otherwise, and regarding psychological well-being, mental health has been considered a very important resource for athletes in relation to their performance and professional development. Recent studies (Gouttebarge et al., 2015) show that more than one-third (38%) of active professional soccer players suffer from depression or a similar disorder, as well as 35% of retired players. Similarly, the probability of a professional suffering depression increases by up to four points when the player has suffered at least three serious injuries, the pressure exerts before the expectations of a large signing and/or an unfulfilled self-demand occurs. In general, athletes experience situations (high training loads, highly relevant competitions, stressful lifestyle) that are real risk factors for their mental health (Schinke et al., 2018). In this regard, the International Society of Sports Psychology (ISSP) has presented six proposals and recommendations to address the mental health of athletes from an intervention and research perspective (Henriksen et al., 2019).

On the other hand, although psychological programs focused on Cognitive-Behavioral Therapy have shown their effectiveness (McArdle and Moore, 2012; Brown and Fletcher, 2017), other types of programs that could be effective in sports have also been proposed in recent years. For instance, in the study by Gross et al. (2018), university athletes who participated in a mindfulness program (Mindfulness-Acceptance-Commitment, MAC) reported reduced anxiety, eating problems, and other psychological disorders; increased psychological flexibility; and had better sport performance than the group of university athletes who participated in a conventional program of psychological skills. As indicated by Bühlmayer et al. (2017), mindfulness as a form of mental training oriented to the present affects cognitive processes and is considered increasingly significant for sport psychological training approaches. In any case, these results, rather than invalidating the Cognitive-Behavioral Therapy programs, present other options for psychological preparation that led by expert psychologists (Aoyagi et al., 2017; Portenga et al., 2017) can complement what already exists.

However, far from affecting only the sports context, the application of these programs can also represent an extraordinary learning for daily life. Learning skills in the sports field can be closely related to learning life skills; for this, it must be transferred and applied successfully beyond sport. As Pierce et al. (2017) stated recently, the transfer of life skills is an essential process that has not yet been sufficiently described in the scientific literature of sports psychology. Therefore, stress control could be a very important application tool from the sports context to the vital (daily life) context of the athlete.

Finally, it is necessary to point out that this work provides evidence on the effectiveness of a cognitive behavioral intervention in youth soccer players, using conventional psychological techniques of confirmed validity, such as visualization (Wesch et al., 2016; Simonsmeier and Buecker, 2017). Epidemiological studies have indicated that sports practice in the youth represents a protective factor against psychological imbalances (Brière et al., 2018); so if psychological work is also available, this protection could be increased. The work of a sport psychologist in these adolescent ages is really relevant for a good sport and social development of the youth athletes, both in the work with the athletes themselves and with coaches and parents (Tjomsland et al., 2016; Lorenzo et al., 2018). These ages constitute a fundamental stage for the acquisition of good practices and habits for a future professional sports career or, simply, a healthy vital relationship with sports.

## Limitations and Future Research Directions

The main limitation of the current research is the small sample size of soccer players (*N* = 19) who received the psychological intervention, which makes it difficult to extrapolate the present results to other cohorts of soccer players. In addition, the participation of the coaches in the study was unequal, showing, sometimes, a lack of involvement in the planning of the program. The increased involvement by these coaches could favor a greater participation of the players in the intervention program, making possible at the same time the application of parallel programs to coaches that could improve the results obtained in the current study. Also, the timing in which the psychological program was carried out (mid-season) hindered its development and made it impossible to compare the effects of the intervention in different sections of the season. Therefore, future investigations should study the possible differences derived from the implementation of this type of psychological program in several competitive phases of the season, using a larger sample size of soccer players and checking the effect of parallel interventions with coaches.

## Conclusions

The findings of this study show that the implementation of a psychological training program of duration 50 min (per session) for eight sessions can be effective to provide psychological skills to youth players that will help them to better manage the stress of sports practice, both in competition and training sessions.

## Data Availability Statement

The datasets generated for this study are available on request to the corresponding author.

## Ethics Statement

This study was carried out in accordance with the recommendations of the Declaration of Helsinki. The studies involving human participants were reviewed and approved by the Comité de Ética de la Universidad de Murcia (ID: UM 1551/2017). Written informed consent to participate in this study was provided by the participants’ legal guardian/next of kin.

## Author Contributions

AO and IM-F contributed to the conception and design of the study. AO and EO organized the database. EO and IV performed the statistical analysis. AO wrote the first draft of the manuscript. IM-F, VG-E, and FR-P wrote the sections of the manuscript. FR-P was in charge of the formal aspects of the work. All authors contributed to the revision of the manuscript and read and approved the presented version.

### Conflict of Interest

The authors declare that the research was conducted in the absence of any commercial or financial relationships that could be construed as a potential conflict of interest.
